# Biological Control of *Meloidogyne incognita* by *Aspergillus niger* F22 Producing Oxalic Acid

**DOI:** 10.1371/journal.pone.0156230

**Published:** 2016-06-03

**Authors:** Ja Yeong Jang, Yong Ho Choi, Teak Soo Shin, Tae Hoon Kim, Kee-Sun Shin, Hae Woong Park, Young Ho Kim, Hun Kim, Gyung Ja Choi, Kyoung Soo Jang, Byeongjin Cha, In Seon Kim, Eul Jae Myung, Jin-Cheol Kim

**Affiliations:** 1 Department of Agricultural Chemistry, Institute of Environmentally Friendly Agriculture, College of Agriculture and Life Sciences, Chonnam National University, Gwangju, Republic of Korea; 2 Department of Plant Medicine, Chungbuk National University, Cheongju, Republic of Korea; 3 Center for Eco-friendly New Materials, Korea Research Institute of Chemical Technology, Daejeon, Republic of Korea; 4 Crop Protection Research Team, Dongbu Advanced Research Institute, Dongbu Farm Hannong Company, Ltd., Nonsan-si, Republic of Korea; 5 Biological Resources Center, Korea Research Institute of Bioscience and Biotechnology, Daejeon, Republic of Korea; 6 World Institute of Kimchi, an Annex of Korea Food Research Institute, Gwangju, Republic of Korea; 7 Department of Agricultural Biotechnology and Research Institute of Agriculture and Life Sciences, Seoul National University, Seoul, Republic of Korea; Woosuk University, REPUBLIC OF KOREA

## Abstract

Restricted usage of chemical nematicides has led to development of environmentally safe alternatives. A culture filtrate of *Aspergillus niger* F22 was highly active against *Meloidogyne incognita* with marked mortality of second-stage juveniles (J2s) and inhibition of egg hatching. The nematicidal component was identified as oxalic acid by organic acid analysis and gas chromatography-mass spectroscopy (GC-MS). Exposure to 2 mmol/L oxalic acid resulted in 100% juvenile mortality at 1 day after treatment and suppressed egg hatching by 95.6% at 7 days after treatment. Oxalic acid showed similar nematicidal activity against *M*. *hapla*, but was not highly toxic to *Bursaphelenchus xylophilus*. The fungus was incubated on solid medium and dried culture was used for preparation of a wettable powder-type (WP) formulation as an active ingredient. Two WP formulations, F22-WP10 (ai 10%) and oxalic acid-WP8 (ai 8%), were prepared using F22 solid culture and oxalic acid. In a field naturally infested with *M*. *incognita*, application of a mixture of F22-WP10 + oxalic acid-WP8 at 1,000- and 500-fold dilutions significantly reduced gall formation on the roots of watermelon plants by 58.8 and 70.7%, respectively, compared to the non-treated control. The disease control efficacy of the mixture of F22-WP10 + oxalic acid-WP8 was significantly higher than that of a chemical nematicide, Sunchungtan (ai 30% fosthiazate). These results suggest that *A*. *niger* F22 can be used as a microbial nematicide for the control of root-knot nematode disease.

## Introduction

Plant disease caused by nematode infection is a major problem in crop production. Plant-parasitic nematodes caused annual yield loss estimated at 8.8–14.6% of total crop production [1]. Root-knot nematodes (RKNs; *Meloidogyne* spp.), scientifically and economically the most important phytonematode [2], occur globally, especially in tropical and sub-tropical agricultural areas, and cause significant yield losses annually [3]. Southern root-knot nematode (*M*. *incognita*), one of the most important species of RKNs, infects the roots of almost all cultivated plants. RKNs impede uptake of water and nutrients due to formation of giant cells in the roots of many crops, and facilitate infection by pathogenic microorganisms.

Control of RKNs is difficult because they have short generation times and high reproduction rates [4, 5]. Several management strategies can be applied to control RKNs; chemical nematicides have generally been used. However, these agents are increasingly being withdrawn or restricted due to their potential negative impact on human health and the environment [6]. In addition, policies designed to support sustainable or environmentally friendly farming practices in many countries and increased demand for eco-friendly measures has necessitated development of safe and effective alternatives [7]. Thus, much research has aimed to identify antagonistic microorganisms and their metabolites with nematicidal activity against RKNs [8–10]. However, this achieved limited success when applied in the field, and consequently few commercial products have been developed and used in agricultural practice [11]. Therefore, identification of antagonists for control of plant parasitic nematodes and their commercialization are necessary.

Microbial agents generally have slower nematicidal activity than chemical nematicides. In certain agricultural applications, slower nematicidal activities have prevented adoption of biopesticides. In addition, the inconsistent performance of microbial agent must be overcome if these are to be used as commercial nematicides [12]. Development of appropriate formulations would facilitate large-scale utilization of biocontrol agents. A suitable formulation benefits the application and handling of the bio-agent, and increases its efficacy by protecting the active ingredient from adverse environmental factors [13, 14]. Although developing appropriate commercial formulations of antagonistic microorganisms is important, a few studies have been reported [15, 16]. Recently, *Bacillus subtilis* strain CPA-8 formulations were prepared by spray drying using various combinations of skimmed milk and MgSO_4_ as carriers/protectants. The CPA-8 formulation (1.6–3.3 × 10^9^ CFU/g) exhibited efficacy similar to fresh cells in terms of controlling brown rot disease [15]. In addition, a wettable powder-type formulation of *Pseudomonas fluorescens* EPS62e using lactose as a lyoprotectant showed improved biocontrol efficacy and survival at low relative humidities [16].

In the course of screening of nematicidal fungi, we found that *Aspergillus* sp. F22 culture filtrate had marked nematicidal activity against *M*. *incognita*. Therefore, the aims of this study were 1) to investigate the nematicidal potential of strain F22, 2) to characterize the nematicidal metabolites produced by the fungus, and 3) to evaluate the efficacy of the strain F22 formulation as a biological control agent for *M*. *incognita* under field conditions.

## Materials and Methods

### Antagonistic fungal strain

*Aspergillus* sp. F22 was isolated from soil on Gyejok Mountain, Daejeon, Korea. Strain F22 was deposited in the Korean Collection for Type Cultures (KCTC) as KCTC12771BP. A stock culture of the strain was stored in 8% dimethyl sulfoxide suspension at -80°C. Strain F22 was maintained on potato dextrose agar (PDA) slants and stored at 4°C. The strain was grown at 25°C on PDA medium, and then inoculated into 500 mL Erlenmeyer flasks containing 200 mL of potato dextrose broth (PDB). Strain F22 was grown at 25°C in PDB for 7 days with shaking at 150 rpm and filtered through sterile gauze to yield culture filtrate.

### Identification of the antagonistic fungal strain

Cells were disrupted by bead beating and DNA extraction was carried out as described by Yu and Mohn [17]. PCR-mediated amplification of the 5.8S-ITS region was carried out using the procedures described by Scorzetti et al. [18]. PCR products were purified using a QIA quick PCR purification kit (Qiagen, Hilden, Germany) and sequenced on an Applied Biosystems 310 DNA sequencer using a Taq DyeDeoxy Terminator cycle sequencing kit (Applied Biosystems, Foster City, CA, USA), according to the manufacturer’s protocol. Existing sequences for reference species were retrieved from GenBank. Sequences were aligned using the multiple sequence alignment software CLUSTAL X and manually corrected [19]. A phylogenetic tree was obtained from the DNA sequences using the neighbor-joining method [20] with Kimura’s two-parameter model for calculating genetic distances [21] and 1,000 bootstrap replications using the PHYLIP 3.57c software [22]. The tree was visualized using the TreeView software [23]. *Talaromyces flavus* NRRL 2098 (EU021596) was used as the designated outgroup.

### Nematodes

*M*. *incognita*, which was isolated and identified by Hwang et al. [24], was maintained on tomato (Lycopersicon esculentum Mill. cv. Seokwang) in a greenhouse (28 ± 5°C). Eggs were extracted from tomato roots infected with *M*. *incognita* using 1% sodium hypochlorite solution. The nematode eggs were collected by passage through a 45 μm sieve, followed by a 25 μm sieve. Collected eggs were rinsed with distilled water and used for *in vitro* experiments and allowed to hatch using modified Baermann funnels [25] at 28°C within 5 days to obtain second-stage juveniles (J2s).

*Meloidogyne hapla* was isolated from root galls of ginseng cultivated in Jinan-gun, Jeon-buk, Korea [26]. Four-week-old tomato plants (*Solanum lycopersicum* cv. Rutgers) grown in a growth chamber were inoculated with J2s of *M*. *hapla* and cultivated at 25±2°C in a greenhouse. J2s of *M*. *hapla* were obtained using the same methods applied to *M*. *incognita*. *B*. *xylophilus* was isolated from chips of infested pine trees and cultured on PDA plates containing *Botrytis cinerea* at 25°C. *B*. *cinerea* were obtained from the Southern Forest Research Center of the Korea Forest Research Institute. Nematodes were extracted using the Baermann funnel method, followed by sieving (38 μm).

### Effect of F22 culture filtrate on *M*. *incognita* J2s and eggs

Effects of *Aspergillus* sp. F22 culture filtrate (1.25–20%) on *M*. *incognita* J2s and eggs were evaluated. Approximately 50 J2s and 150 eggs per well were used for the bioassay using 96-well tissue culture plates (Becton Dickinson, Franklin Lakes, NJ). Sterile distilled water (SDW) was used as the negative control. The plates were gently shaken and incubated at 100% humidity in a plastic box at room temperature in the dark. All experiments were conducted with three replicates and the experiment was twice repeated. J2s were counted after 1 day of incubation and judged as dead if their body was straight with no movement despite physical stimulation with a fine needle. Mortality rates (M) were corrected using Abbott’s formula [27]:

$$\text{M}=\left[\frac{\left(\text{Mt}-\text{Mc}\right)}{\left(100-\text{Mc}\right)}\right]\times 100$$

Where Mt means mortality percentage in treatment and Mc means mortality percentage in control. The rate of inhibition of nematode egg hatching was assessed under a light microscope at 7 days after treatment. Hatch inhibition (HI) was calculated according to the formula:

$$\text{HI}=\left[\frac{\left(\text{C }-\text{ T}\right)}{\text{C}}\right]\times 100$$

Where C and T are the percentages of eggs hatched in the control and treatment, respectively. Egg hatch rate (EH) was calculated as follows:

$$\text{EH}=\left[\frac{\text{J}}{\left(\text{E }+\text{ J}\right)}\right]\times 100$$

Where J and E mean the juveniles and eggs of *M*. *incognita*, respectively.

### Organic acid analysis

*Aspergillus sp*. F22 culture filtrate was passed through a membrane filter (0.45 μm). The filtrate was diluted fivefold and organic acids were analyzed by high-performance liquid chromatography (HPLC) (Agilent 1100 series HPLC, Santa Clara, CA, USA). An Aminex HPX-87C column (4.6 × 250 mm, Bio-Rad, Marnes-la-Coquette, France) was used for analysis. Elution was carried out isocratically using 5 mmol/L sulfuric acid. The flow rate and column temperature were 0.6 mL/min and 28°C, respectively. Detection was performed by a UV detector at 210 nm (G1314A, Agilent HPLC 1100 series). Quantitative analysis of organic acids was performed using standard curves.

To confirm the presence of oxalic acid, the filtrate was further analyzed by GC-MS after methyl derivatization. An aliquot (0.5 mL) of culture filtrate was mixed with 0.5 mL of 29 mmol/L malonic acid, and then added to 2 mL of methanol and 0.4 mL of 50% sulfuric acid (H_2_SO_4_). The sample was heated at 60°C for 30 min with caps closed and then 1 mL of distilled water and 0.5 mL of chloroform added, followed by agitation for 30 s. The chloroform layer was taken and 1 μL of the sample was injected into the GC-MS. GC-MS analysis was conducted using an Agilent Technologies system (Wilmington, DE, USA) consisting of a model 7890 gas chromatograph and a model 5975 mass selective detector. The column was a DB-WAX (id 0.25 mm × length 30 m, film thickness 0.25 μm). GC temperature program was as follows: initial temperature of 40°C, which was held for 1 min, increased to 150°C at a rate of 2°C/min, then to 200°C at a rate of 4°C/min, and finally to 250°C at a rate of 6°C/min, and held for 10 min. The split ratio was 1:12, injection temperature was 200°C, transfer line temperature was 250°C, and ion source temperature was 200°C. The mass spectrometer was operated at 70eV in the electron impact mode. The spectrum obtained from the sample was identified by comparison with mass spectra in the NIST library and a standard solution (0.1 mol/L oxalic acid).

### Effects of oxalic acid, citric acid and tartaric acid on *M*. *incognita* J2s and eggs

Stock solutions of oxalic acid (≥99% purity; Sigma-Aldrich, St. Louis, MO), citric acid (99% purity; Sigma-Aldrich), and tartaric acid (99% purity; Sigma-Aldrich) were prepared using distilled water. A stock solution of *trans*-cinnamaldehyde (cinnamaldehyde; 99% purity; Sigma-Aldrich), which is used as a natural nematicide [28], was prepared using ethanol. Stock solutions were tested at a concentration range of 0.08–50 mmol/L. The final concentration of ethanol did not exceed 1% of the volume. Ethanol (1%) was used as a negative control. Ninety- or ninety-nine-microliter aliquots of suspensions of J2s (~50 per well) or eggs (~150 per well) were placed in each well, followed by addition of 10 μL of stock solutions of organic acids or 1 μL of stock solution of cinnamaldehyde. The juvenile mortality and egg hatching rates were evaluated as described above. The experiment was carried out with five replicates and the experiment was twice repeated. *M*. *incognita* juvenile mortality and egg hatching rates were determined after 1 and 7 days of incubation, respectively.

### Effect of oxalic acid on J2s of *M*. *hapla* and *B*. *xylophilus*

The nematicidal activity of oxalic acid (0.4–10 mmol/L) was investigated against *M*. *hapla* and *B*. *xylophilus* as described above for *M*. *incognita*. Distilled water (10%) was used as the negative control. Ninety-microliter aliquots of juvenile suspension (~50 per well) were placed in each well, followed by addition of 10 μL of stock solution. The experiment was carried out with three replicates and the experiment was twice repeated. The mortality rate of juvenile nematodes was evaluated after 1 day of incubation.

### Solid-state fermentation of *Aspergillus* sp. F22 and formulation

The fungal strain F22 was cultured on PDA at 25°C for 7 days and then spores were harvested using distilled water (1.0 × 10^6^ spores/mL). Sterile wheat-rice bran medium (wheat bran 100 g, rice bran 100 g, and distilled water 100 mL in a 1 L Erlenmeyer flask) was inoculated with 2 mL of spore suspension, followed by incubation at 30°C for 7 days in the dark to yield 4.5 × 10^9^ spores/g. Solid-state culture of *Aspergillus* sp. F22 (100g, 10%) was mixed with 720 g of kaoline, 90 g of sodium dodecyl sulfate (CR-SDS; Yoosung Chemical R&T Co., Ltd, Daejeon, Korea) as a wetting agent, and 90 g of sodium poly(naphthalene formaldehyde) sulfonate (CR-100; Yoosung Chemical R&T Co., Ltd) as a dispersal agent. The wettable powder-type formulation of *Aspergillus* sp. F22 (F22-WP10) was milled in a blender (IKA^®^-Werke GmbH & Co. KG, Staufen, Germary). The *A*. *niger* F22 formulation had viable spore count of 4.1 × 10^8^ CFU/g.

Oxalic acid (95% purity) was purchased from Chunbo Fine Chemical (Eumseong-gun, Korea). A wettable powder-type formulation of oxalic acid (OA-WP8) was prepared by mixing 80 g of oxalic acid, 270 g of synthetic hydrated silicon dioxide (white carbon; Rhodia Asia Pacific Pte Ltd, The Concourse, Singapore), 90 g of CR-SDS, 90 g of CR-100, and 570 g of kaoline [29].

### Efficacy of F22 for biocontrol of *M*. *incognita* on watermelon plants under field conditions

The experiment was conducted in a field naturally infected with RKNs (*Meloidogyne* spp.), located in Chaeun-myeon, Nonsan-si, Chungnam, Korea in June 2015. The owner of the land gave permission to conduct the study on this site. The field was severely affected by RKNs during previous seasons. Watermelon seeds (cv. Uriggul) were sown into seed trays containing clay-loam soil in a greenhouse and after 3 weeks transplanted into the plots. To calculate the initial nematode density in the soil, five soil cores (250 cm^3^ each) were collected from each plot before transplantation of watermelon. The nematode densities in soil were assessed by soil core sampling (7.2 × 10 cm) at a depth range of 10–20 cm per treatment plot selected randomly. Nematodes in soil were extracted from a 200 g soil subsample by sieving and centrifugation [30] and 4 × 3 mL aliquots of a 50 mL suspension counted on a counting dish under a microscope (Olympus CX31). The initial nematode densities were 235 ± 16 J2s and 55 ± 3 eggs per 100 mL soil. The field experiment was performed using a randomized complete block design with four replicates; each treatment consisted of 10 m^2^ (2 × 5 m^2^) plots. The treatments were: (1) untreated control, (2) 1,000- and (3) 500-fold dilutions of F22-WP10, (4) 500-fold dilution of OA-WP8, (5) 1,000- and (6) 500-fold dilutions of a mixture of F22-WP10 and OA-WP8, and (7) 2,000-fold dilution of Sunchungtan (EC: ai 30% fosthiazate, 70% surfactant; Dongbu Farm Hannong Co., Korea). An aliquot (200 mL) of each treatment was applied to the soil around watermelon roots following the start of gall formation four times at 10-day intervals. The negative control was 200 mL of water only. At 60 days after the first treatment, the galling index (GI) was assessed according to a 0–5 galling scale: 0 = 0–10% galled roots, 1 = 11–20%, 2 = 21–50%, 3 = 51–80%, 4 = 81–90%, and 5 = 91–100% [31]. The control value was calculated according to the formula: control value (%) = 100 × (galling index of control—galling index of treatment) / galling index of control.

### Statistical Analysis

Data were subjected to one-way ANOVA and the means of the treatments were separated by Duncan’s multiple range test (*p* < 0.05) using the SPSS software (SPSS, version 21.0 for Windows, Chicago, IL). The 50% effective concentrations (EC_50_) were determined by probit analysis (95% confidence limits) using the SPSS software.

## Results

### *In vitro* nematicidal activity and identification of F22

**Fig 1:**
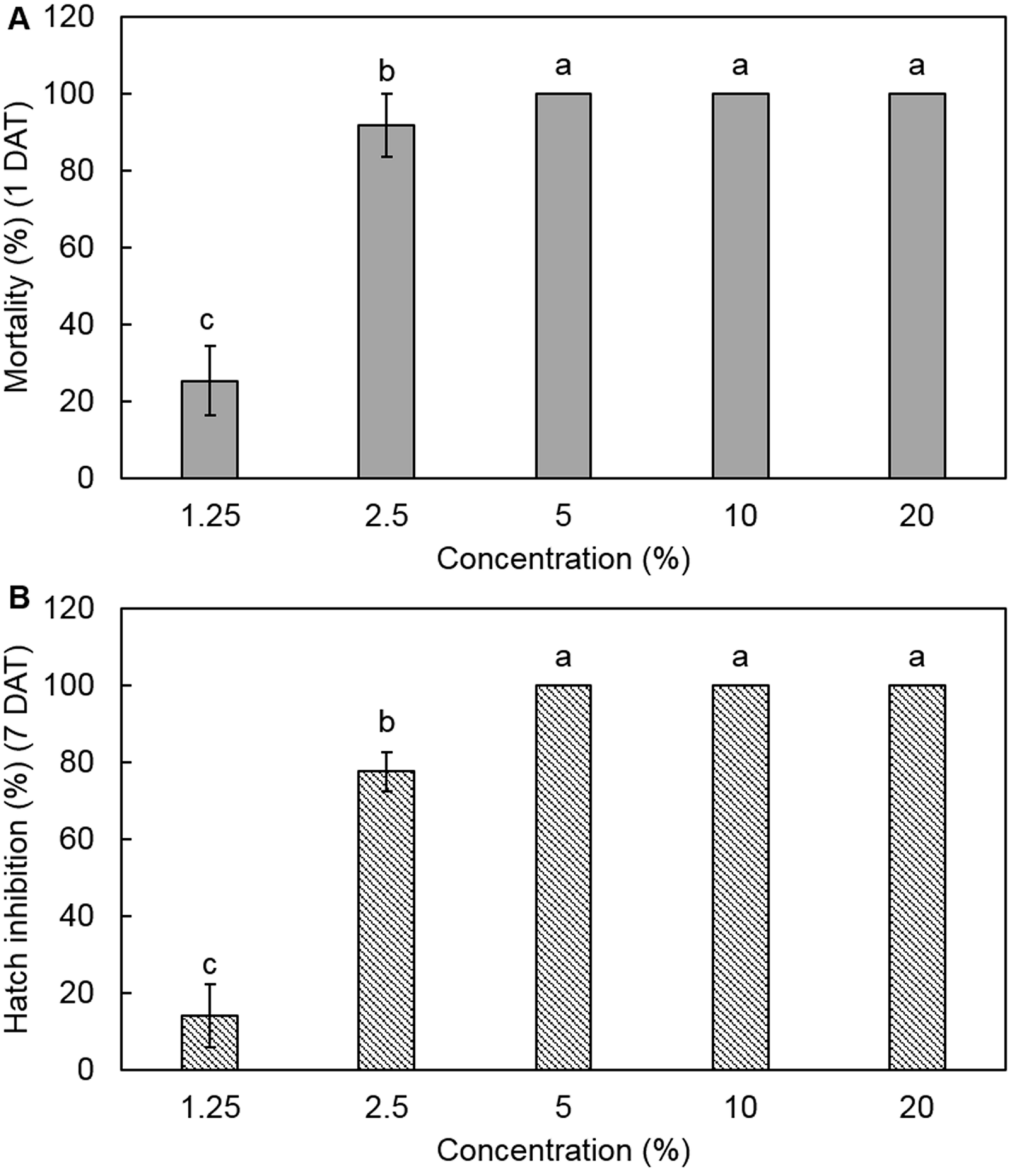
Effects of F22 culture filtrate on J2 mortality (A) and egg hatching (B) of *M*. *incognita*. Values are means ± standard deviation of three replicates. Means with the same letter are not significantly different (*p* < 0.05) according to Duncan’s multiple range test.

The culture filtrate of strain F22 at various concentrations showed nematicidal activity against J2s and inhibited egg hatching. Its effects on killing J2s and suppressing egg hatching increased in a dose-dependent manner. The J2 mortality rate was 25.3% at 1.25% culture filtrate and >90% at 2.5–20% culture filtrate at 1 day after treatment (Fig 1A). Complete inhibition of egg hatching was observed at 5–20% culture filtrate after 7 days of exposure as compared to the control (Fig 1B). The rate of inhibition of egg hatching was reduced to 77.5% and 14.0% at 2.5% and 1.25% culture filtrate, respectively. Fungal strain F22 exhibited 98.7% similarity to *Aspergillus niger* CBS 554.65^T^ (AJ223852) according to 5.8S-ITS rRNA gene sequence analysis (S1 Fig). The F22 sequence was deposited in GenBank under accession number KU221232.

### Identification of the nematicidal metabolite

To identify the nematicidal metabolite produced by strain F22, organic acid analysis was conducted because the culture filtrate showed strong acidity (pH 1.7). Oxalic aicd (7.9 g/L) was the major organic acid in culture filtrate of strain F22 (Table 1). Citric acid and tartaric acid were also detected, albeit at lower concentrations (461.6 and 388.1 mg/L, respectively) than that of oxalic acid. Based on its content in culture filtrate and its nematicidal activity, OA was identified as a major nematicidal component. GC-MS analysis after methyl derivatization confirmed the presence of oxalic acid in the F22 culture filtrate. The EI-mass spectrum of oxalic acid methyl ester showed a molecular ion peak at *m/z* 118 [M^+^] and a base peak at *m/z* 59 [M-COOCH_3_^+^] (S2 Fig), which was identical to that of the standard compound.

**Table 1:** Production of organic acids by *A*. *niger* F22 in potato dextrose broth medium.

Organic acid	Production (mg/L)
Acetic acid	ND ^a^
Citric acid	461.6
Fumaric acid	5.4
Lactic acid	ND
Malic acid	ND
Oxalic acid	7928.4
Succinic acid	ND
Tartaric acid	388.1

^a^ ND: not detected.

### Effects of oxalic acid, citric acid, and tartaric acid on J2s and eggs of *M*. *incognita*

**Fig 2:**
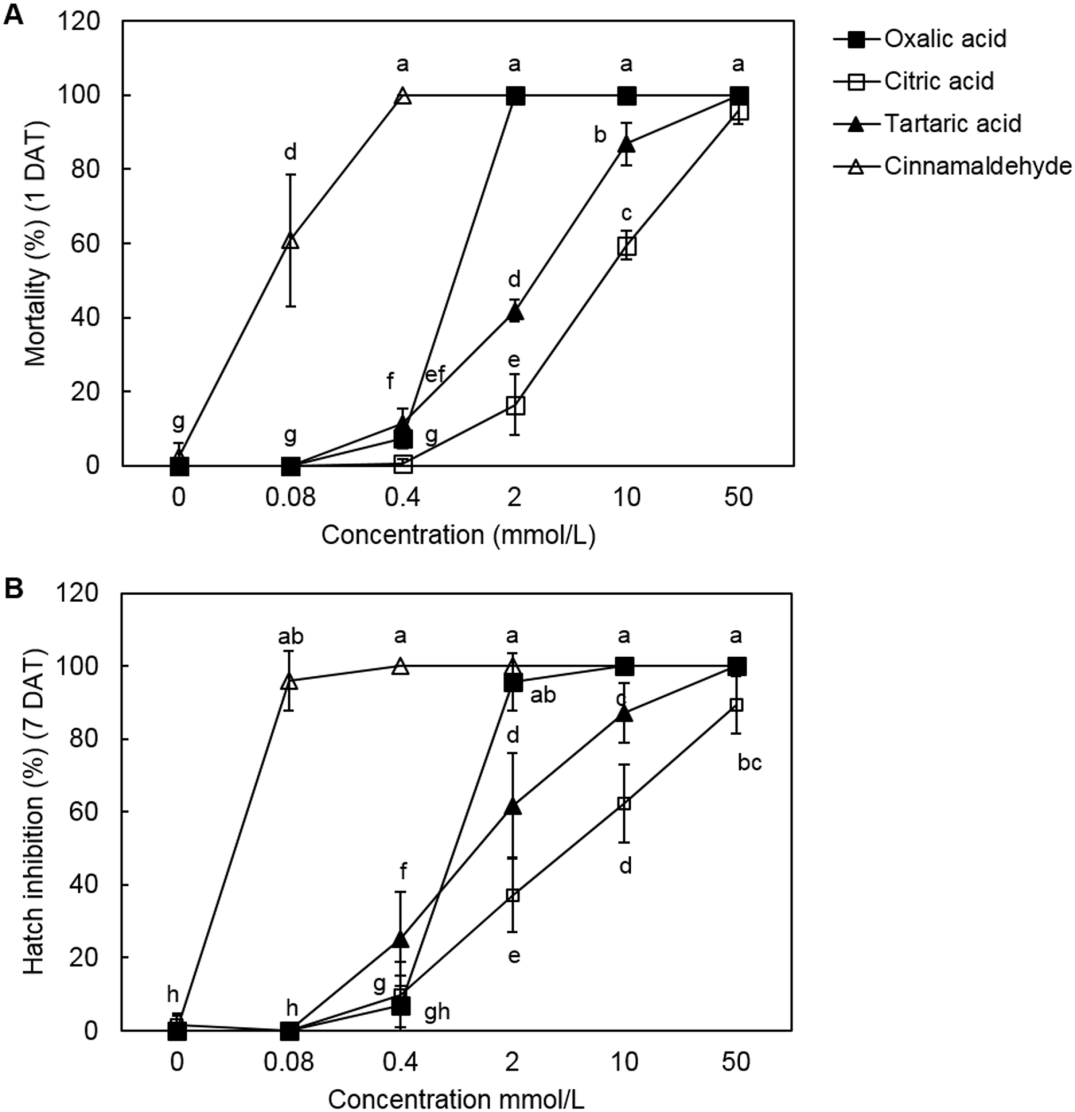
Effects of oxalic acid, citric acid, tartaric acid and cinnamaldehyde on *M*. *incognita* J2 mortality and egg hatching rates. Values are means ± SD of five replicates. Means with the same letter are not significantly different (*p* < 0.05) according to Duncan’s multiple range test.

Exposure to 2 mmol/L oxalic acid solution caused 100% mortality of *M*. *incognita* J2s at 1 day after treatment (Fig 2A). The nematicidal activity of oxalic acid against *M*. *incognita* J2s was lower than that of cinnamaldehyde, a natural nematicide. Exposure of *M*. *incognita* J2s to 50 mmol/L citric acid and tartaric acid resulted in 96.0% mortality, whereas application of 10 mmol/L resulted in mortality rates of 59.5% and 86.9%, respectively. The EC_50_ values of oxalic acid and cinnamaldehyde were 0.87 and 0.17 mmol/L, respectively. The EC_50_ values of citric acid and tartaric acid were 14.35 and 4.32 mmol/L, respectively, at 1 day after treatment.

**Fig 3:**
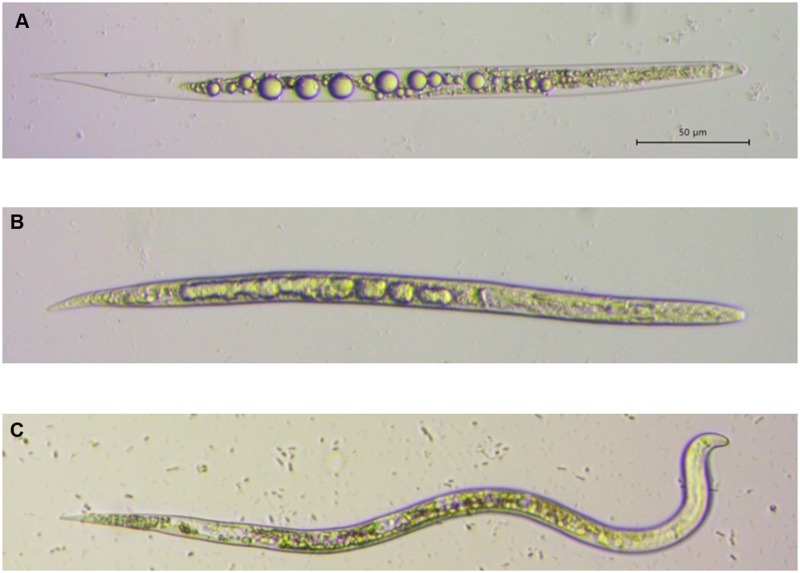
Morphological variations in *M*. *incognita* J2s treated with 2 mmol/L oxalic acid (A), 2 mmol/L cinnamaldehyde (B), and water (C). Bar = 50 μm.

In addition, treatment with 2 mmol/L oxalic acid resulted in 95.6% inhibition of egg hatching. Moreover, 10 and 50 mmol/L oxalic acid resulted in 100% inhibition of egg hatching at 7 days after treatment (Fig 2B). In comparison, 0.4 mmol/L cinnamaldehyde completely inhibited egg hatching. Treatment with 50 and 10 mmol/L citric acid and tartaric acid resulted in 89.3% and 100%, 62.4% and 87.2%, inhibition of egg hatching, respectively. The EC_50_ values of oxalic acid, citric acid, tartaric acid, and cinnamaldehyde were 1.15, 17.08, 4.20, and 0.16 mmol/L, respectively, at 7 days. Microscopic observation indicated that OA oxalic acid completely destroyed internal organs, resulting in production of multiple vacuoles in the nematode body, which was straightened and stiffened (Fig 3). In comparison, the exposure to cinnamaldehyde at the same concentration caused relatively weak disruption of internal organs. The negative control (1% ethanol) did not induce such symptoms in *M*. *incognita* J2s.

**Fig 4:**
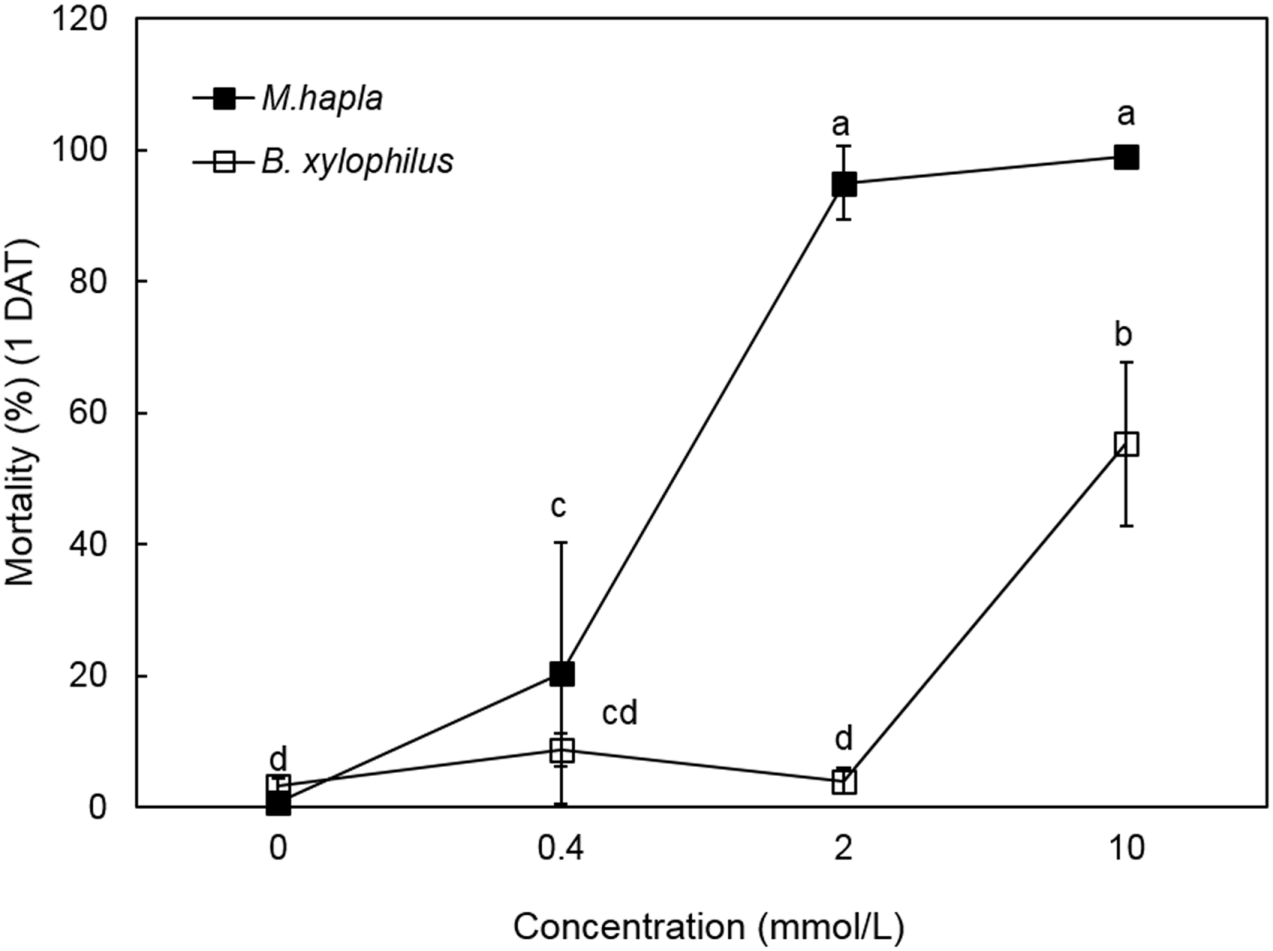
Oxalic acid -induced mortality of *M*. *hapla* and *B*. *xylophilus* J2s. Values are means ± SD of three replicates. Means with the same letter are not significantly different (*p* < 0.05) according to Duncan’s multiple range test.

In addition, the nematicidal activity of oxalic acid against J2s of *M*. *hapla* and *B*. *xylophilus* was investigated. As shown in Fig 4, *M*. *hapla* was highly sensitive to oxalic acid, to a level similar to *M*. *incognita*, but *B*. *xylophilus* was relatively resistant compared to the two *Meloidogyne* species. The EC_50_ values of oxalic acid were 1.15 mmol/L for *M*. *hapla* and 9.37 mmol/L for *B*. *xylophilus*.

### Biocontrol of root knot nematode disease by F22 under field conditions

**Fig 5:**
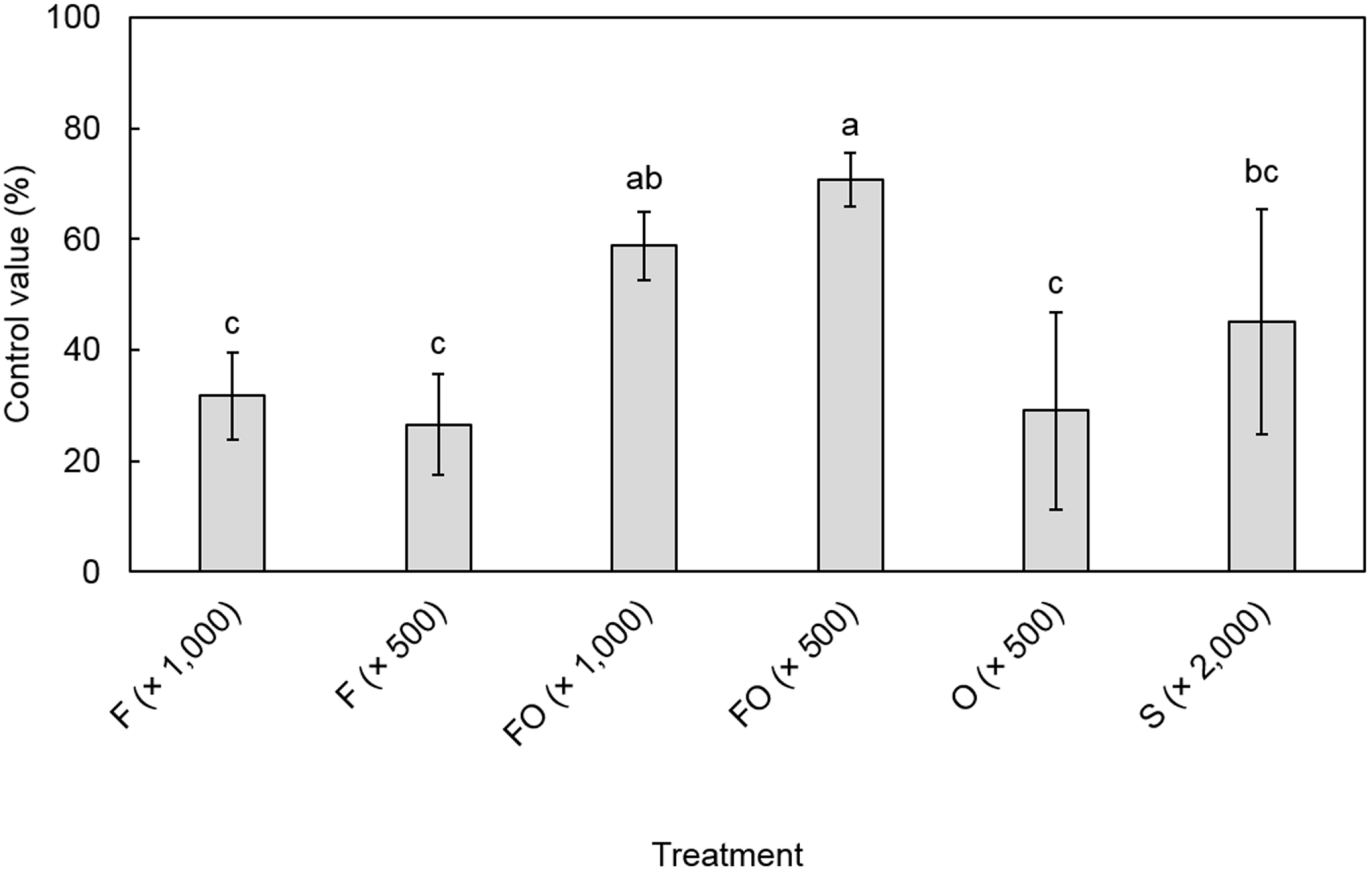
Efficacy of agents individually or in combination on watermelon plants (cv. Uriggule) in a field naturally infected condition by *M*. *incognita*. F (×1,000), 1,000-fold dilution of F22-WP10; F (×500), 500-fold dilution; FO, F22-WP10 + OA-WP8; O, OA-WP8; and S (× 2,000), 2,000 fold-dilution of Sunchungtan (150 μg/mL fosthiazate). F22-WP10: a wettable powder-type formulation of *A*. *niger* F22 (ai 10%), OA-WP8: a wettable powder-type formulation of oxalic acid (ai 8%). Data are means ± SD of four replicates. Relationships among values were subjected to one-way ANOVA and Duncan’s multiple range test. Means with the same letter did not differ significantly (*p* < 0.05).

Under field conditions, F22-WP10 and OA-WP8, at a 500-fold dilution, resulted in moderate inhibition of gall formation on the roots of watermelon plants (Fig 5). Co-treatment with the two formulations resulted in significantly higher inhibitory activity than those of either formulation alone. Application of a 1:1 mixture of the two formulations, at 1,000- and 500-fold dilutions, significantly (*p*<0.05) reduced gall formation by 58.8% and 70.7%, respectively. This activity was higher than that of Sunchungtan (45.1%), a chemical nematicide.

## Discussion

Sustainable pest management in agriculture is hampered by nematodes, especially in intensive greenhouse cultivation [32]. Prohibition of the use of common chemical nematicides and their decreased efficacy due to continuous utilization are barriers to control of RKNs [33]. Moreover, global warming has conditions favorable for RKNs. Identification of environmentally friendly and non-toxic control measures of RKNs is thus required. Our results indicated that exposure to *A*. *niger* F22 culture filtrate reduced the rates of J2 viability and egg hatching. This effect was attributed to the production of oxalic acid as a nematicidal metabolite. Mankau (1969) reported oxalic acid to be a nematicidal metabolite of *A*. *niger* and its nematicidal effect both *in vitro* and in soil against *Aphelenchus avenae* [34]. Kurt et al. [35] reported that *A*. *niger* cause moderate toxicity against *Aphelenchus avenae* and *A*. *besseyi* by producing oxalic acid and citric acid. Zuckerman et al. [36] reported that both citric acid and oxalic acid were nematicidal metabolites in culture filtrate from an isolate of *A*. *niger* (designated PD-42), but their nematicidal effects against *Caenorhabditis elegans* were weak; >10 g/L oxalic acid resulted in 100% mortality of *C*. *elegans* juveniles, but 16 g/L citric acid did not show 100% mortality. However, the combination of oxalic acid (4 g/L) and citric acid (4 g/L) was nematicidal, suggesting a synergistic effect. Furthermore, Mokbel et al. [37] reported that culture filtrate of *A*. *niger* and oxalic acid and citric acid have nematicidal activity against *M*. *arenaria* as a similar result. In this study, we report that oxalic acid produced by *A*. *niger* F22 has strong nematicidal activity against *M*. *incognita* and *M*. *hapla*.

The nematicidal activity of low-molecular-weight organic acids has been reported [38–40]. Browing et al. [38] reported that exposure to 0.1 and 1 mol/L butyric acid resulted in 100% mortality of *M*. *hapla* and *M*. *incognita*. Seo and Kim [39] reported that a mixture of acetic acid and lactic acid had greater toxicity on *M*. *incognita* J2s than either agent alone. Also, Bansal and Bajaj [40] reported that six volatile fatty acids reduced the rate of egg hatching during 12 days of incubation in the following order: propionic acid > acetic acid > caprylic acid > isobutyric acid > valeric acid > butyric acid. To our knowledge, this is the first report of nematicidal activity and egg hatching inhibitory activity of oxalic acid against *M*. *incognita*. Although the mode of action of oxalic acid is unknown, the strong acidity of oxalic acid (pKa = 1.25) may cause rapid destruction of cells and tissues of the nematode bodies and eggs [39]. This may be caused by osmoregulation disruption and then fluid accumulation. Our microscopic analysis supported these hypotheses (Fig 3). We investigated pH values of three organic acids and cinnamaldehyde. Except cinnamaldehyde, as the concentration of the three organic acids in solutions increased, the more acidic the solutions became (S3 Fig). At the same concentrations, pH values of oxalic acid were highest, followed in order by tartaric acid and citric acid. This trend coincided with the extents of mortalities of three organic acids; the mortality of oxalic acid was highest among the three organic acids, followed in order by tartaric acid and citric acid (Fig 2). These results strongly suggest that the nematicidal activity of oxalic acid is closely related to its strong acidity. However, the other mechanism should not be ruled out completely. *A*. *niger* F22 in liquid medium produces oxalic acid, citric acid and tartaric acid. Compared to oxalic acid, citric acid and tartaric acid were not highly toxic to *M*. *incognita*. Thus oxalic acid was a major nematicidal component of F22 culture filtrate. Oxalic acid is present in various foods and plants (such as amaranth, cassava, chives, and parsley) at levels of >1.0 g/100 g [41]. Oxalic acid is used rarely in Canada, Europe, and the USA as a biopesticide for the control of varroa mite on honey bees [42–43]. Therefore, *A*. *niger* F22 culture filtrate and oxalic acid can be used as biopesticides for the control of RKNs.

In the field experiment, treatment of soil with the F22-WP10 + OA-WP8 mixture resulted in greater efficacy than either agent alone. The treatment of F22-WP + OA-WP8 mixture not only may rapidly reduce the *M*. *incognita* J2 population density, but may also maintain a low nematode population through production of oxalic acid by *A*. *niger* F22. On the other hand, Gadd [44] reported that oxalic acid and citric acid produced by fungi may bind to metal ions including Zn^++^, and it suppressed egg hatching due to interaction with Zn^++^ ions [45]. However, the underlying mechanism is unknown. Also, oxalic acid would likely induce systemic resistance in plants against diseases caused by fungi, bacteria, and viruses and may enhance host defense by increasing defense-related enzyme activities and production of secondary metabolites, such as phenolics [46–49]. This may hinder nematode invasion and subsequent infection. Zuckerman et al. [36] reported that incorporation of *A*. *niger* PD-42 in seed coats suppressed gall formation on pepper by *M*. *incognita* and increased yield. These previous reports as well as our results suggest the possibility of commercial use of the mixture of *A*. *niger* F22 solid culture and oxalic acid in farming. This is the first study of a commercial *A*. *niger* and oxalic acid formulation for control of *M*. *incognita*.

Because of the issue of pesticide residue, Sunchungtan (ai. fosthiazate 30%) is generally used once before plantation, and it cannot be used during crop cultivation. Although the pesticide was treated four times at 10-day intervals in this study, its disease control efficacy against RKN disease in watermelon was significantly lower than that of a 500-fold dilution of a 1:1 mixture of F22-WP10 + OA-WP8. This suggests that the F22-WP10 + OA-WP8 mixture would show high disease control efficacy in the field. In addition, the mixture of F22-WP10 + OA-WP8 can be used during crop cultivation. This will facilitate effective control of RKNs in the field. Even though *A*.*niger* has been generally regarded as safe, it have the potential for mycotoxin production such as fumonisins and ochratoxins [50]. Therefore, we need to scrutinize its production potential before commercialization.

Biopesticides comprise a small proportion of the pesticides used for crop protection. Biological products are highly target-specific and their use is desirable, but acceptable formulations are difficult to develop [51]. The formulated product must be stored under appropriate conditions prior to application for maximum efficacy. Our results suggest the mixture of F22-WP10 and OA-WP8 to be a promising nematicide for the control of RKNs in conventional farming systems. Further studies are required 1) to examine the toxicity of the two formulations, 2) to evaluate the disease control efficacy of the mixture of the two formulations in various fields, 3) to examine the disease control spectrum of the two formulations against various nematode diseases, and 4) to develop more effective and eco-friendly formulations.

## Supporting Information

S1 Fig Neighbour-joining phylogenetic tree based on 5.8S-ITS sequences of F22 and its closest relatives. Numbers at nodes represent the proportions of 1,000 bootstrap samples in which a clade was found. Bar, 0.01 substitutions per nucleotide position. *Talaromyces flavus* NRRL 2098 was used as the outgroup.
(TIF)

S2 Fig GC-EI-MS total ion chromatogram of *Aspergillus niger* F22 culture filtrate (A) and mass spectrum of the peak at the retention time 25.347 (B). Standard solution was prepared at a concentration of 0.1 mol/L oxalic acid.
(TIF)

S3 Fig pH values of oxalic acid, citric acid, tartaric acid, and cinnamaldehyde with different concentrations. Values are means ± standard deviation of three replicates.
(TIF)
