# Supplementary figures and images for: Biological Control of Meloidogyne incognita by Aspergillus niger F22 Producing Oxalic Acid
Source: PLoS One. 2016 Jun 3;11(6):e0156230. doi: 10.1371/journal.pone.0156230 (PMC4892604; doi:10.1371/journal.pone.0156230)

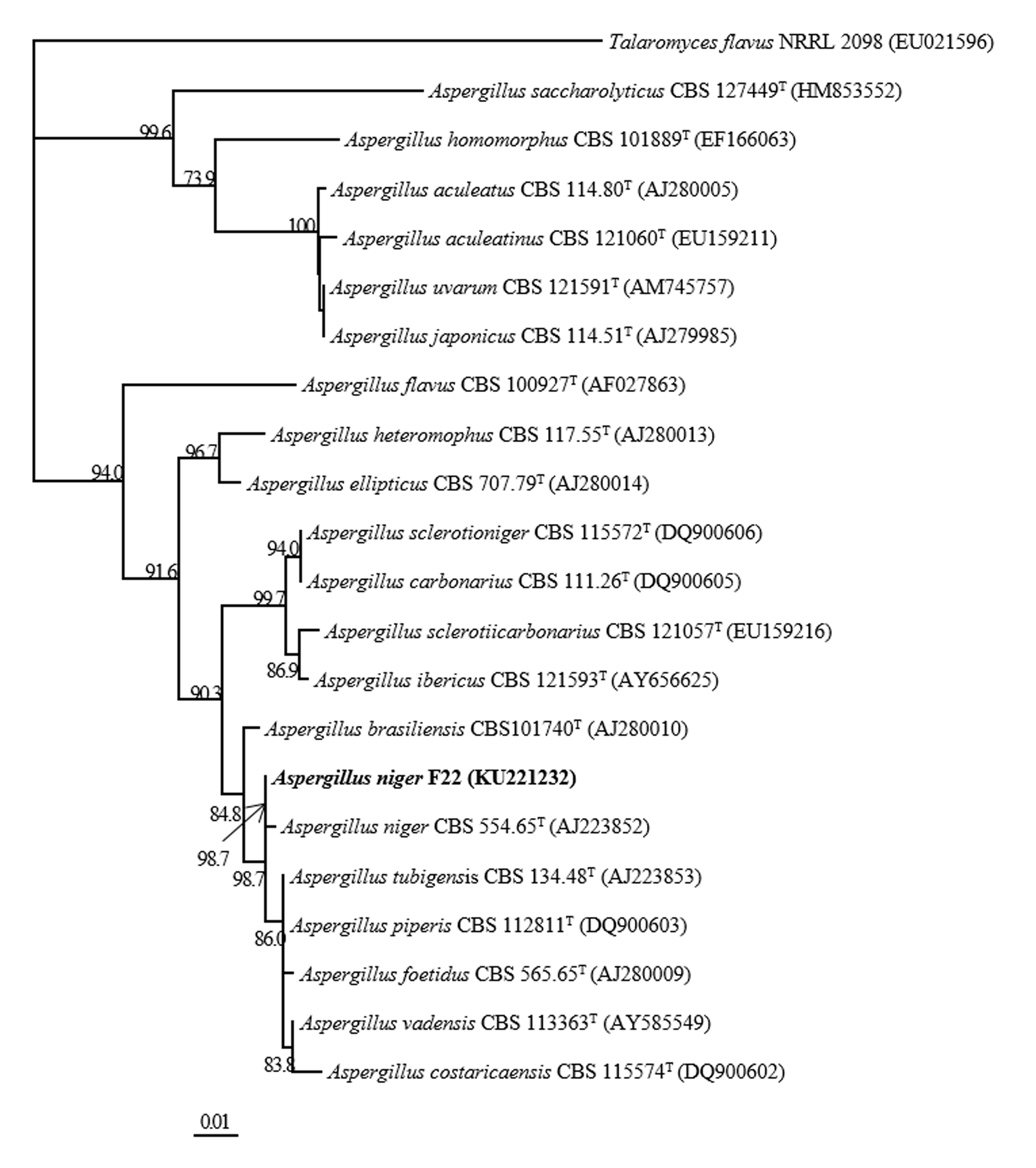

Supplement: S1 Fig — Numbers at nodes represent the proportions of 1,000 bootstrap samples in which a clade was found. Bar, 0.01 substitutions per nucleotide position. Talaromyces flavus NRRL 2098 was used as the outgroup. (TIF) [file pone.0156230.s001.tif]

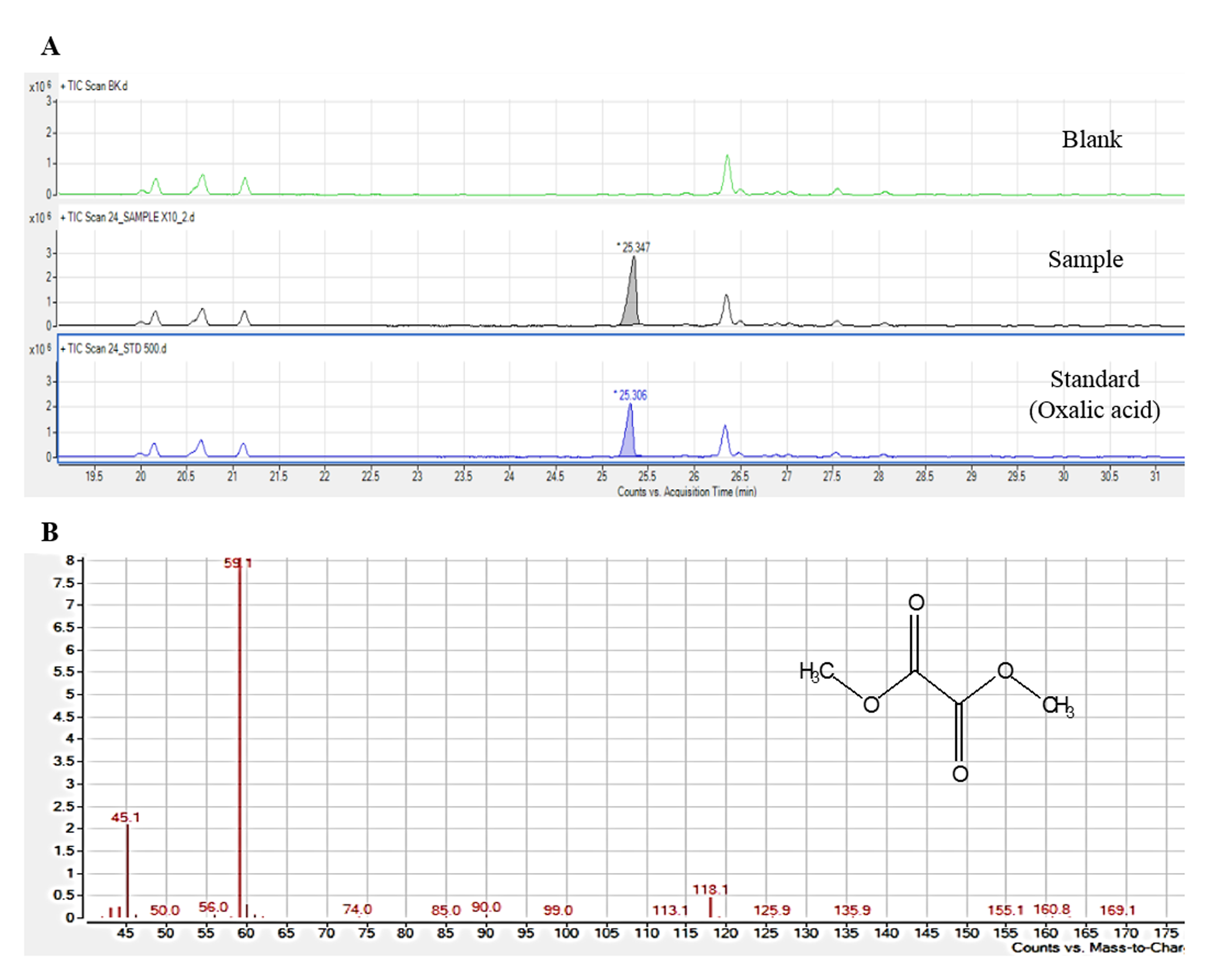

Supplement: S2 Fig — Standard solution was prepared at a concentration of 0.1 mol/L oxalic acid. (TIF) [file pone.0156230.s002.tif]

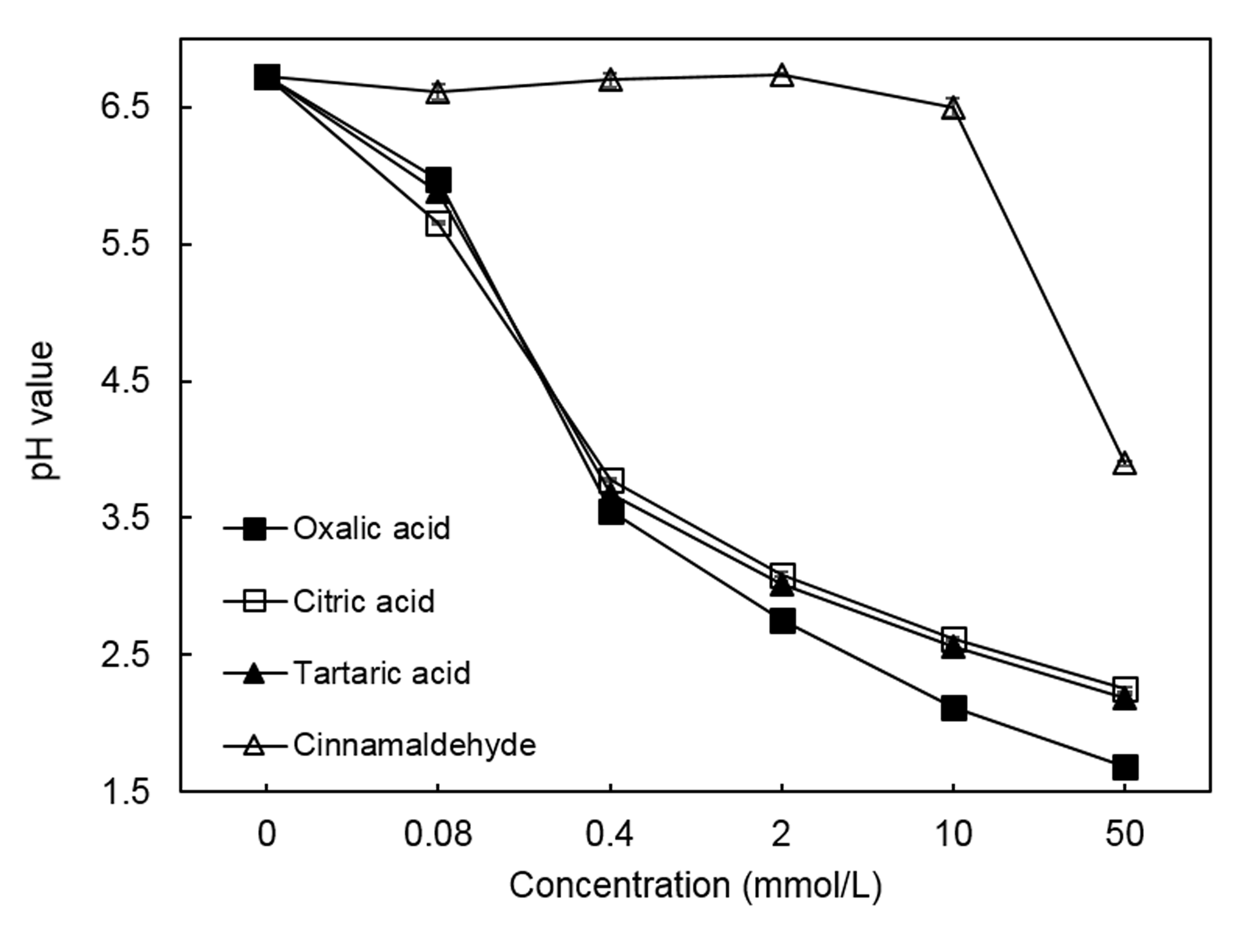

Supplement: S3 Fig — Values are means ± standard deviation of three replicates. (TIF) [file pone.0156230.s003.tif]
